# Multi-Omics Approach Reveals Prebiotic and Potential Antioxidant Effects of Essential Oils from the Mediterranean Diet on Cardiometabolic Disorder Using Humanized Gnotobiotic Mice

**DOI:** 10.3390/antiox12081643

**Published:** 2023-08-20

**Authors:** María José Sánchez-Quintero, Josué Delgado, Laura Martín Chaves, Dina Medina-Vera, Mora Murri, Víctor M. Becerra-Muñoz, Mario Estévez, María G. Crespo-Leiro, Guillermo Paz López, Andrés González-Jiménez, Juan A. G. Ranea, María Isabel Queipo-Ortuño, Isaac Plaza-Andrades, Jorge Rodríguez-Capitán, Francisco Javier Pavón-Morón, Manuel F. Jiménez-Navarro

**Affiliations:** 1 Biomedical Research Institute of Malaga and Nanomedicine Platform (IBIMA Plataforma BIONAND), 29590 Málaga, Spain; mj.sanchez@ibima.eu (M.J.S.-Q.); lauramartinchaves@gmail.com (L.M.C.); dina.medina@ibima.eu (D.M.-V.); moramurri@gmail.com (M.M.); vmbecerram@gmail.com (V.M.B.-M.); iqueipo@uma.es (M.I.Q.-O.); isaacplazaandrade@gmail.com (I.P.-A.); mjimeneznavarro@uma.es (M.F.J.-N.); 2 Heart Area, Hospital Universitario Virgen de la Victoria, 29010 Málaga, Spain; 3 Biomedical Research Network Center for Cardiovascular Diseases (CIBERCV), Instituto de Salud Carlos III, 28029 Madrid, Spain; marisacrespo@gmail.com; 4 Higiene y Salud Alimentaria, Faculty of Veterinary, University of Extremadura, 10003 Cáceres, Spain; jdperon@unex.es; 5 Instituto Universitario de Investigación de Carne y Productos Cárnicos (IPROCAR), University of Extremadura, 10003 Cáceres, Spain; mariovet@unex.es; 6 Department of Dermatology and Medicine, Faculty of Medicine, University of Málaga, 29010 Málaga, Spain; 7 Clinical Management Unit of Mental Health, Hospital Regional Universitario de Málaga, 29010 Málaga, Spain; 8 Clinical Management Unit of Endocrinology and Nutrition, Hospital Universitario Virgen de la Victoria, 29010 Málaga, Spain; 9 Biomedical Research Network Center for the Physiopathology of Obesity and Nutrition (CIBEROBN), Instituto de Salud Carlos III, 28029 Madrid, Spain; 10 Service of Cardiology, Complexo Hospitalario Universitario A Coruña (CHUAC), University of A Coruña, Instituto Investigación Biomédica A Coruña (INIBIC), 15006 A Coruña, Spain; 11 Bioinformatics, Common Support Structures (ECAI), IBIMA Plataforma BIONAND, 29590 Málaga, Spain; guillepl@uma.es (G.P.L.); bioinformatica@ibima.eu (A.G.-J.); ranea@uma.es (J.A.G.R.); 12 Department of Molecular Biology and Biochemistry, Faculty of Science, University of Málaga, 29010 Málaga, Spain; 13 CIBER of Rare Diseases (CIBERER), Instituto de Salud Carlos III, 28029 Madrid, Spain; 14 Intercenter Clinical Management Unit of Medical Oncology, Hospitales Universitarios Regional y Virgen de la Victoria y Centro de Investigaciones Médico Sanitarias (CIMES), 29010 Málaga, Spain; 15 Department of Surgical Specialties, Biochemistry, and Immunology, Faculty of Medicine, University of Málaga, 29010 Málaga, Spain

**Keywords:** antioxidant, carbonyl, coronary artery disease, diabetes mellitus, essential oil, Mediterranean diet, microbiota, omics, oregano, thyme

## Abstract

Essential oils sourced from herbs commonly used in the Mediterranean diet have demonstrated advantageous attributes as nutraceuticals and prebiotics within a model of severe cardiometabolic disorder. The primary objective of this study was to assess the influences exerted by essential oils derived from thyme (*Thymus vulgaris*) and oregano (*Origanum vulgare*) via a comprehensive multi-omics approach within a gnotobiotic murine model featuring colonic microbiota acquired from patients diagnosed with coronary artery disease (CAD) and type-2 diabetes mellitus (T2DM). Our findings demonstrated prebiotic and potential antioxidant effects elicited by these essential oils. We observed a substantial increase in the relative abundance of the *Lactobacillus* genus in the gut microbiota, accompanied by higher levels of short-chain fatty acids and a reduction in trimethylamine N-oxide levels and protein oxidation in the plasma. Moreover, functional enrichment analysis of the cardiac tissue proteome unveiled an over-representation of pathways related to mitochondrial function, oxidative stress, and cardiac contraction. These findings provide compelling evidence of the prebiotic and antioxidant actions of thyme- and oregano-derived essential oils, which extend to cardiac function. These results encourage further investigation into the promising utility of essential oils derived from herbs commonly used in the Mediterranean diet as potential nutraceutical interventions for mitigating chronic diseases linked to CAD and T2DM.

## 1. Introduction

Cardiovascular diseases stand as the foremost among non-communicable diseases on a global scale. Traditional pharmacological interventions impart a diverse spectrum of side effects in patients [1]. Hence, the pursuit of alternative therapeutic compounds takes precedence. The mounting body of evidence suggests that appropriate nutrition may be a pivotal factor in cardiovascular disease prevention. Moreover, the inclusion of micronutrients and nutraceuticals is linked to a marked enhancement in disease progression among patients [2,3].

In recent years, a plethora of studies have examined the potential therapeutic attributes of essential oils and intricate metabolites displaying pharmacological traits, such as antibacterial, antifungal, anti-inflammatory, and antioxidant activities, among others [4]. Several studies have elucidated the capacity of distinct plant-derived essential oils to enhance the cardiovascular system, fostering vasorelaxation, and attenuating the atherosclerosis process [1]. Furthermore, these oils serve as prebiotic agents, antioxidants, immunomodulators, and anti-inflammatory mediators [5,6,7,8].

Cardiovascular and metabolic diseases have been linked to gut microbiota dysbiosis, a condition susceptible to modification through dietary changes and susceptible to detrimental effects from antibiotics or food additives [9]. The composition and activity of the microbial population can be also influenced by essential oils, which promote the proliferation of specific beneficial microorganisms [5,10]. The presence of commensal bacteria in the gastrointestinal tract is closely connected to the generation of metabolites of paramount importance in preserving human health, such as trimethylamine (TMA) and short-chain fatty acids (SCFAs) [11,12,13,14]. Essential oils possess the potential to bolster the prevalence of advantageous microorganisms, such as *Lactobacillus* spp., while diminishing bacterial populations that fail to confer benefits or are linked to metabolic disturbances [10].

Additionally, certain essential oils have been documented to exhibit antioxidant effects [15], functioning as scavengers that target reactive oxygen species (ROS) and mitigate oxidative stress. Amid pathological conditions, these radical and non-radical entities, arising from the partial reduction in oxygen, are endogenously generated in mitochondrial oxidative phosphorylation [16]. Mitochondria play a critical role in the cardiac function since the cardiac mitochondria occupy nearly one-third the volume of a cardiomyocyte [17]. However, although mitochondria consume the most amount of oxygen and several forms of ROS are generated, additional organelles and cellular compartments are also implicated, attributable to a complex interplay of cellular processes. Notably, ROS has recently been proposed as a novel integrated network for sensing homeostasis and alarming stress within metabolic pathways across various subcellular organelles [18].

Metabolically, oxidative stress results in direct or indirect ROS-mediated damage of nucleic acids, lipids, and proteins. Thus, oxidized proteins and glycoproteins have been used as markers in various chronic diseases characterized by enduring oxidative stress, including renal failure and diabetes, both common cardiovascular comorbidities [19,20]. Among oxidized proteins, alpha-aminoadipic semialdehyde (AAS) and gamma-glutamic semialdehyde (GGS) stand out as principal carbonyl products, while pentosidine represents an advanced glycation end product (AGE) resulting from the combined processes of glycosylation and oxidation. Specific natural products and plant-derived compounds have demonstrated antioxidant effects by reducing the levels of these metabolic end products, thereby presenting potential therapeutic attributes against cardiometabolic disorders [15,21].

Essential oils have been commercially explored as additives, food preservatives, and diet supplements [22]; however, their interplay with the metabolic pathways involved in the progression of cardiometabolic disorders remains unknown [10]. Consequently, a deeper investigation into the therapeutic potential of essential oils as nutraceuticals at the molecular and cellular level, encompassing varied outcomes and employing diverse tissues and techniques, becomes essential.

The objective of this study was to assess the impacts of essential oils derived from thyme (*Thymus vulgaris*) and oregano (*Origanum vulgare*), two herbs commonly featured in the Mediterranean diet, using a gnotobiotic murine model harboring colonic microbiota extracted from patients diagnosed with coronary artery disease (CAD) and type 2 diabetes mellitus (T2DM). To unravel the metabolic effects and mechanisms of action, a comprehensive multi-omics approach was conducted. This approach encompassed analysis of the metagenomic and proteomic changes linked to the administration of these essential oils, in conjunction with an exploration of their metabolic and antioxidant potential.

## 2. Materials and Methods

### 2.1. Ethical Statement and Animals

This study received approval from the Animal Experimentation Ethics Committee of IBIMA Plataforma BIONAND (code 23/10/2018/151) in accordance with the European directive 2010/63/EU, designed to protect animals used in scientific research and adhering to the Spanish regulations for the care and utilization of laboratory animals (RD53/2013 and RD118/2021). The protocols and procedures were executed in compliance with the ARRIVE (Animal Research: Reporting of In Vivo Experiments) guidelines, with diligent efforts made to minimize animal suffering.

CD1 male mice (Janvier Labs, Le Genest-Saint-Isle, France) were allowed to acclimate over a period of 4 weeks, in accordance with the recommendations and guidelines stipulated by the Animal Experimentation Facility of IBIMA Plataforma BIONAND. The duration of this acclimation period was determined based on a range of factors, including stress and variability reduction, normalization of physiological parameters (in response to changes in diet, lighting, and housing conditions), familiarization of the mice to human handling, facilitation of behavioral and social adaptation, addressing ethical concerns within our facility, and drawing from prior research) [15].

The mice were accommodated within a vivarium maintaining controlled humidity and temperature. They were subjected to a 12 h light–dark cycle with food (Table S1) and water ad libitum.

### 2.2. Humanized Gnotobiotic Mouse Model

Thirty CD1 mice were employed to establish a humanized gnotobiotic mouse model as previously described [15,23,24,25,26,27]. Briefly, the animals were treated with antibiotics for 10 consecutive days to reduce the diversity of the gut microbial community. Subsequently, the mice were inoculated for three consecutive days with fecal microbiota from selected patients to recolonize their gastrointestinal tract via oral gavage. The fecal samples were previously procured from high-risk CAD patients which specific characteristics detailed in Alcoholado et al. [28]. For the inoculation process, a pooled sample was prepared mixing aliquots from these patients, ensuring a uniform distribution of human microbiota across all animals. The cages were cleaned every two days to prevent the possibility of reinoculation from old fecal material.

### 2.3. Essential Oil Composition and Preparation of Essential Oil Emulsions and Vehicle

Commercially available food-grade essential oils extracted from thyme (*Thymus vulgaris*) and oregano (*Origanum vulgare*) were purchased from Farmacia Rico Néstares (Málaga, Spain). The chemical profiling of the essential oils was determined by solid-phase microextraction coupled with gas chromatography-mass spectrometry (SPME-GC-MS) using a gas chromatograph 6890 GC (Agilent Technologies, Santa Clara, CA, USA) equipped with an HP-5 column (5% phenyl-95% dimethylpolysiloxane: 30 m × 0.25 mm × 0.25 μm) and a mass spectrometer detector, 5975C (Agilent Technologies, Santa Clara, CA, USA). The carrier gas was helium. The injector port operated in splitless mode and at 250 °C and the injection volume of 1 μL. The temperature program was isothermal for 2 min at 40 °C, followed by an incremental rise at a rate of 5 °C/min to reach 300 °C, where it was held for 5 min. The GC/MS transfer line temperature was set at 280 °C. The mass spectrometer operated in the electron impact mode, with an electron energy of 70 eV, a multiplier voltage of 1650 V, and data collection at a scan rate of 1 scan/s across the m/z range of 50–400. Chemical compounds were identified by comparing their mass spectra to the Wiley and the NIST/EPA/NIH mass spectrum libraries. Chromatographic peaks areas were quantified and reported in arbitrary area units. The composition of both essential oils is provided in Table S2.

The essential oils were diluted in distilled water with lecithin (1.2% *w*/*v*) and maltodextrin (24% *w*/*v*) to create an emulsion for oral administration at two different concentrations, 0.3 and 0.6% *w*/*v*, corresponding to doses of 10 mg/kg and 20 mg/kg. A vehicle solution, comprising water with lecithin and maltodextrin, was formulated for the control group.

### 2.4. Design and Experimental Groups

After fecal microbiota transplantation, 20-week-old male mice were exposed to drinking water supplemented with 0.02% (*v*/*w*) L-carnitine during 40 consecutive days. Additionally, essential oils or vehicle were administered daily by gavage in a final volume of 100 µL. The dosages were established as previously reported [29], with an initial basal dosage of 10 mg/kg/day. Subsequently, this basal dosage was increased to 20 mg/kg/day to investigate potential dose-dependent effects of the essential oils.

The mice were randomly assigned to five experimental groups, each comprising six animals per group (*n* = 6): (1) Control group, receiving L-carnitine supplementation and vehicle treatment; (2) Thyme-10 group, receiving L-carnitine supplementation and thyme essential oil treatment at a concentration of 10 mg/kg/day; (3) Thyme-20 group, receiving L-carnitine supplementation and thyme essential oil treatment at a concentration of 20 mg/kg/day; (4) Oregano-10 group, receiving L-carnitine supplementation and oregano essential oil treatment at a concentration of 10 mg/kg/day; (5) Oregano-20 group, receiving L-carnitine supplementation and oregano essential oil treatment at a concentration of 20 mg/kg/day.

### 2.5. Metagenomic Analysis of Gut Microbiota in Feces

To assess the impact of essential oil treatments on diversity and bacterial populations, we conducted a metagenomic analysis following a methodology previously described [15]. Briefly, gut microbiota was extracted from fecal samples and, subsequently, genomic microbial DNA was amplified using two primer pools designed for the V2 and V3 regions of the bacterial 16S rRNA gene. We created barcoded libraries, and amplicon libraries were subjected to template preparation. Sequencing was executed on an Ion 520 chip using the Ion S5TM System (Thermo Fisher Scientific, Waltham, MA, USA). Alpha and beta diversity of bacterial microbiota in fecal samples were assessed using both the Shannon index and principal components analysis (PCA), respectively.

### 2.6. Determination of SCFA Levels in Feces

We assessed the levels of acetic, propionic, and butyric acid in fecal samples obtained from animals across all the studied groups. Initially, feces were resuspended in 3 mL of double deionized water and hexane 1:1 (*v*/*v*), vortexed and sonicated for 5 min. Then, samples were centrifuged at 3500× *g* for 5 min and the small volume from the upper phase was injected into a 6890 N flame ionization detector gas chromatograph system, equipped with a DB-WAX 60 m × 0.32 mm × 0.25 µm column (Agilent Technologies, Santa Clara, CA, USA). The temperature settings for the injector and detector were set at 100 °C and gradually raised to reach 250 °C over a 15-minute interval. The results were expressed as area under the curve.

### 2.7. Proteomic Analysis of Cardiac Tissues

Proteomic analyses were conducted on cardiac tissue samples collected from all experimental groups. Abundance profiles for peptides from each animal were subjected to mass spectrometry (MS), and the biochemical properties and biological functions of the identified proteins were further analyzed using bioinformatics. Briefly, after 40 days of the treatments with essential oils or vehicle, the animals were euthanized, blood samples were collected, and the heart was carefully dissected. Adjacent fat deposits, auricles, blood vessels, and heart valves were meticulously removed, leaving the ventricle preserved in a protease inhibitor buffer until protein extraction. The tissue was mechanically disintegrated using lysis buffer supplemented with a protease inhibitor cocktail (Sigma-Aldrich, St. Louis, MO, USA) using a TissueLyser (2 × 1 min at 30 Hz) (Qiagen, Hilden, Germany). The final protein concentration was quantified using Qubit fluorometric quantitation (Thermo Fisher Scientific, Waltham, MA, USA). Subsequently, a total of 50 μL of protein extract at a concentration of 2 μg/μL was digested with trypsin, and label-free relative quantification was conducted.

The identification of peptides was carried out using an Easy nLC 1220 UHPLC system coupled with a linear quadrupole-trap hybrid mass spectrometer-Orbitrap Q-exactive HF-X (Thermo Fisher Scientific, Waltham, MA, USA). The software employed for the acquisition of mass spectrum data included Tune 2.9 and Xcalibur 4.1.31.9. A thorough MS/MS2 spectrum search was conducted against the NCBI protein database of *Mus musculus*, and the acquired raw data were analyzed using the Proteome Discover 2.3 platform (Thermo Fisher Scientific, Waltham, MA, USA).

Only proteins that were consistently present in all biological replicates were considered for analysis. The obtained data were normalized by calculating the intensity of each protein divided by the sum of intensities of all proteins in one sample, followed by multiplication by 100. Enrichment analyses for Gene Ontology (GO) and *Kyoto Encyclopedia of Genes and Genomes* (KEGG) were conducted on proteins within the predicted interactions. A fold-change threshold of +/− 0.5 and a significance cutoff of an adjusted *p*-value below 0.05 were applied to identify discriminant proteins. To further elucidate the biological context, proteins were subjected to Enrichr (https://maayanlab.cloud/Enrichr/) (accessed on 15 March 2023) an integrative web software application that includes gene set libraries and synthesized information about hundreds of thousands of mammalian genes and gene sets [30,31,32]. The GeneCards database (http://www.genecards.org/) (accessed on 10 April 2023) was employed to gather relevant information related to genes and to identify the biological function of their proteins.

### 2.8. Determination of Carbonyls and Pentosidine in Plasma

AAS, GGS, and pentosidine were determined in plasma from mice following protocols previously published [15,33]. Briefly, samples were incubated with a freshly prepared solution composed of 0.5 mL of 250 mM 2-(N-morpholino) ethanesulfonic acid (MES) buffer, pH 6.0, containing 1 mM diethylenetriaminepentaacetic acid (DTPA); 0.5 mL of 50 mM 4-amino benzoic acid (ABA) in 250 mM MES buffer pH 6.0 and 0.25 mL of 100 mM sodium cyanoborohydride in 250 mM MES buffer pH 6.0. Subsequently, 50 µL of the samples were processed, and HPLC analysis was conducted. A total volume of 1 μL was injected for analysis, and AAS-ABA, GGS-ABA, and pentosidine were eluted. The identification of derivatized semialdehydes in the fluorescence detector chromatograms was conducted by comparing their retention times with the standard compounds. Results were expressed as nmol per mg of protein.

### 2.9. Determination of Metabolites Related to Cardiovascular Health in Plasma

Trimethylamine N-oxide (TMAO) and cholesterol (total cholesterol and LDL cholesterol) were also determined in plasma to examine supplementary indicators of cardiovascular risk.

For TMAO quantification, a total volume of 15 µL of plasma was incubated with 45 µL of cold methanol for 2 h at −80 °C. After incubation, samples were centrifuged at 18,200× *g* for 12 min at 4 °C, and supernatant was collected and stored at −80 °C for further analysis. TMAO levels were quantified using a Dionex UltiMate 3000 RSLC system coupled to Q Exactive Hybrid Quadrupole-Orbitrap Mass Spectrometer (Thermo Fisher Scientific, Walthman, MA, USA). An Accucore HILIC 150 mm × 2.1 mm × 2.6 µm column was used as stationary phase, while as a mobile phase we used as solvent (A: H_2_O with 0.005 M ammonium formate pH 4.88; and B: acetonitrile and H_2_O (9:1) with 0.005 M ammonium formate pH 4.9). The gradient was isocratic 70% (A/B), the injection volume was 5 µL, and the flow rate was set as 0.4 mL/min. The detection of TMAO was performed with positive ionization in full scan with 70,000 full width at half maximum (FWHM), using the ions *m*/*z* 76.0757 as target and setting 5 parts per million (ppm) of accuracy. Retention time was 2.03 ± 0.1 min for TMAO. Serial dilutions of commercial standards for TMAO from Sigma-Aldrich (St. Louis, MO, USA) were used to determine the linearity between the area under the curve and the concentrations (µM).

Plasma levels of total cholesterol and LDL cholesterol were determined using enzymatic colorimetric kits (a SP41023 Cholesterol-LQ kit based on a CHO-PAP method with reactions catalyzed by cholesterol esterase, oxidase, and peroxidase; and a TK41021 LDLc-D kit based on a two-step method with elimination of lipoprotein no LDL and subsequent measurement of LDL cholesterol) according to the manufacturer’s instructions (Spinreact, San Esteve de Bas, Spain). The results were expressed as mg/dL.

### 2.10. Statistical Analysis

Data in the graphs are expressed as percentages, mean and standard error of the mean (mean ± SEM), and/or median and interquartile range (IQR). All determinations in feces, cardiac tissue, and plasma samples were performed in five treatment groups (the control, oregano-10, oregano-20, thyme-10, and thyme-20 groups), and the statistical analysis of the biochemical data was performed using the Graph-Pad Prism version 5.04 software (GraphPad Software, San Diego, CA, USA). The distribution of raw data from biochemical determinations was assessed in order to determine whether to use parametric or non-parametric tests. Differences in normal variables were assessed using one-way analysis of variance (ANOVA) (F-statistic value is indicated), while non-normal variables were assessed using the Kruskal–Wallis rank sum test (KW statistic values are indicated). The Sidak’s correction test was used as post hoc tests for multiple comparisons in the ANOVA and Kruskal–Wallis rank sum test.

Bacterial sequencing files were analyzed using the Linux system and bash commands, the QIIME 2 platform (version 2022.2.1), within the QIIME 2 command-line interface (q2cli, version 2022.2.0), and the R software environment (version 4.1.3). A quality control step was applied to the sequences. Subsequently, QIIME 2 was used for the identification of ASVs and taxonomic groups. Sequences were filtered and identification and count of ASVs were performed using the DADA2 package (version 2022.2.0) following the QIIME 2 Moving Pictures tutorial. Afterwards, once in R, the sum of ASVs was made by sample and by identified taxa. The results were transformed using the log-ratio transformations function, and the centered log-ratio (CLR) option, from the mixOmics package (version 6.18.1). MicrobiomeAnalyst 2.0 tool was used to perform exploratory analysis of the abundance and the alpha and beta diversity. Differential statistical tests were applied to the transformed data at the genus level to identify significant differences between the study groups. The Kruskal–Wallis test and the Wilcoxon rank sum test, as post hoc test, were used in this step. A significance level of 0.05 was the one considered for the adjusted *p*-value obtained through the false discovery rate method.

## 3. Results

### 3.1. Alterations in Bacterial Abundance of Gut Microbiota Following Essential Oil Treatments

**Figure 1:**
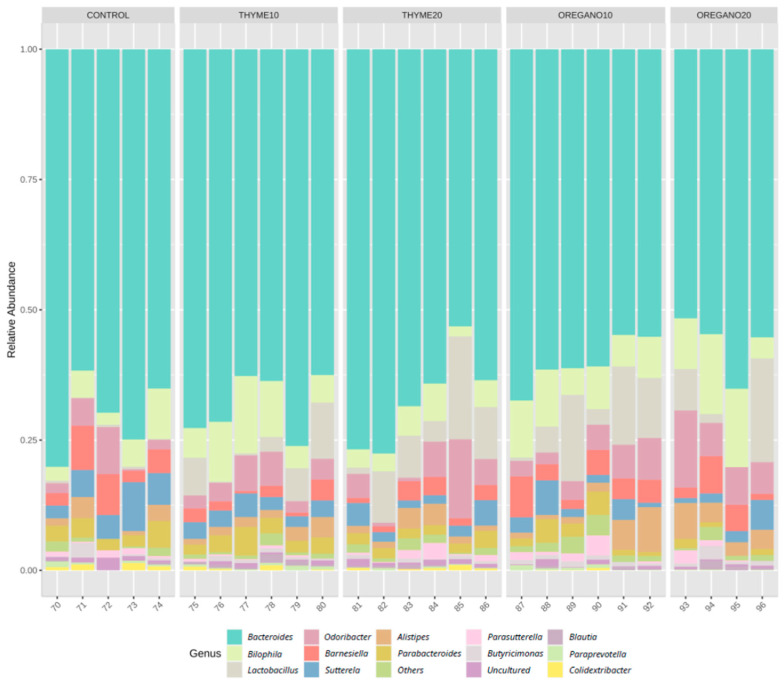
Bacterial abundance profile at the order level in fecal samples of mice from the treatment groups. The bars depict the relative abundances (%) for each group based on 16S rRNA gene sequencing (Ion S5TM System).

**Figure 2:**
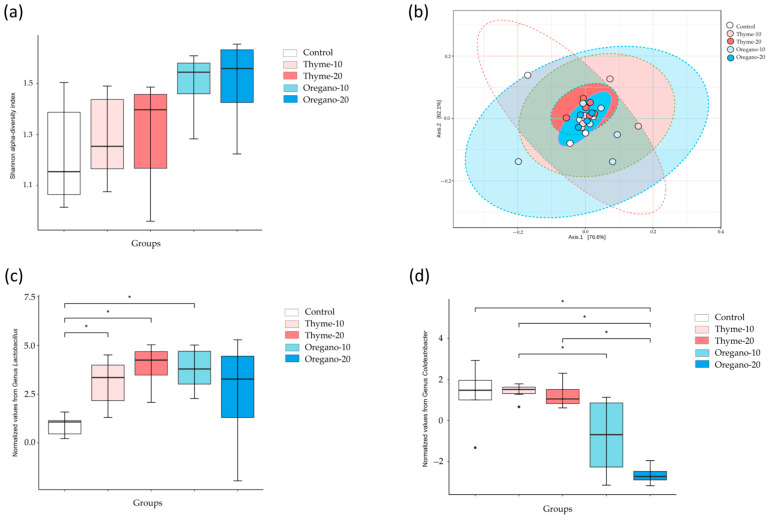
Analyses of alpha and beta diversity of bacterial microbiota in fecal samples from the treatment groups. (**a**) Shannon index; (**b**) Principal components analysis (PCA); (**c**) Differential analysis of the abundance of *Lactobacillus*; (**d**) Differential analysis of the abundance of *Colidextribacter* in fecal samples of mice from the treatment groups. The bars represent median and IQR. Data from the thyme-10, thyme-20, oregano-10, and oregano-20 groups were analyzed using the Kruskal–Wallis test followed by Wilcoxon rank sum test as post hoc test. (*) *p* < 0.05 indicates significant differences compared to the control group.

We performed metagenomics analysis to identify the diversity and taxonomic changes in fecal samples of mice from the experimental groups. The relative abundance of the most abundant genus is represented in Figure 1. Alpha diversity at the genus level, measured as the Shannon index, showed higher diversity in the oregano-10 and oregano-20 groups compared to control mice (Figure 2a). Moreover, beta diversity analyses indicated an increase in total species diversity in mice treated with essential oils derived from thyme and oregano compared to vehicle-treated control mice (Figure 2b). Additionally, the oregano-20 group showed the highest distance to the control group. Diversity analyses were performed to analyze the relative abundances, showing relevant differences among the essential oil groups. At the genus level, *Lactobacillus* was the most predominant genus after the essential oil treatments compared to control mice (*p* = 0.029 for thyme-10; *p* = 0.021 for thyme-20; and *p* = 0.021 for oregano-10) (Figure 2c). Furthermore, there was a lower abundance of *Colidextribacter* genus in the oregano-20 group compared to the control group (*p* = 0.039) (Figure 2d).

### 3.2. Elevated SCFA Levels Confirm the Favorable Impact of Essential Oils on Gut Microbiota

**Figure 3:**
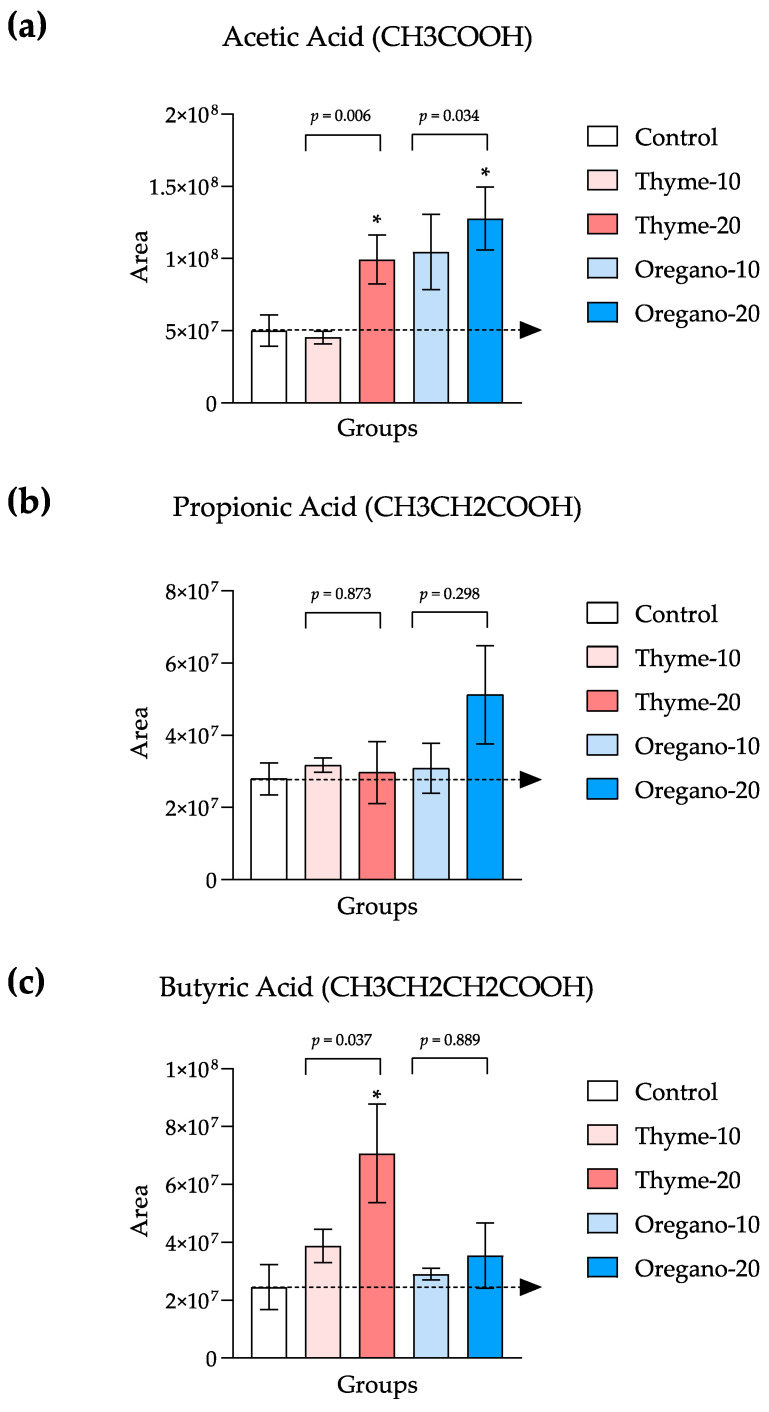
Levels of short-chain fatty acid (SCFA) species in fecal samples of mice from the treatment groups. (**a**) Acetic; (**b**) Propionic; (**c**) Butyric acid levels. The bars represent means ± SEM of SFCA levels (area in arbitrary units). Data were analyzed using one-way ANOVA or the Kruskal–Wallis test, followed by Dunn’s multiple comparison test as post hoc test. (*) *p* < 0.05 indicates significant differences compared to the control group. The black arrow indicates control levels.

To validate the positive impacts of thyme- and oregano-derived essential oils on the gut microbiota, we quantified the levels of SCFAs in the fecal samples of treated mice. The Kruskal–Wallis test revealed significant main effects for both thyme- and oregano-derived essential oil treatments on fecal levels of acetic acid (KW = 8.735 and *p* = 0.006, and KW = 6.268 and *p* = 0.034, respectively) as well as a main effect for the thyme-derived essential oil treatment on butyric acid levels (KW = 6.195 and *p* = 0.037). Subsequent post hoc comparisons demonstrated significant increases in acetic acid levels in the thyme-20 and oregano-20 groups when compared to the control group (*p* < 0.05 for both groups) (Figure 3a) and a significant increase in butyric acid levels in the thyme-20 group when compared to the control group (*p* < 0.05) (Figure 3c).

### 3.3. Over-Representation of Pathways Associated with Cardiac Contraction and Mitochondrial Function in Lower Abundance

To gain a comprehensive understanding of the underlying mechanisms impacted by the administration of essential oils, we conducted a rigorous quality control analysis to identify discriminant proteins across all the experimental groups.

**Figure 4:**
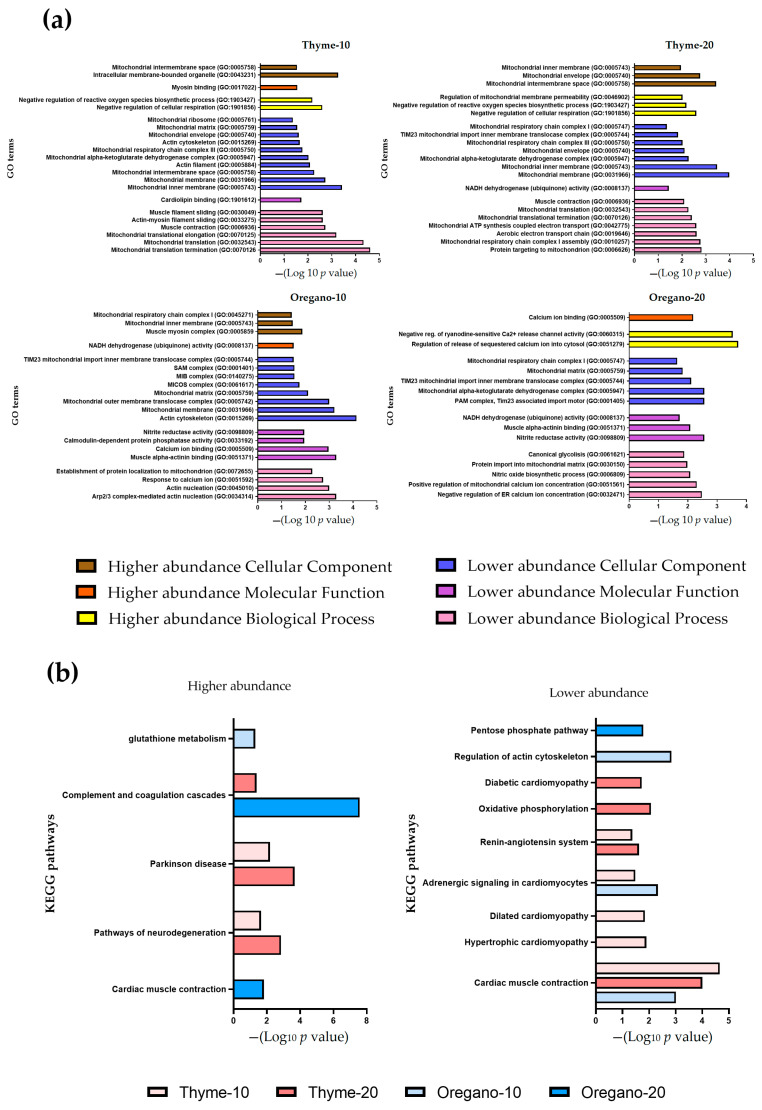
Proteomic analysis in cardiac tissue samples from the treatment groups. (**a**) Gene Ontology (GO) enrichment analysis of higher and lower abundant proteins. Different colors represent each category: biological process, cellular component, and molecular function; the size of each dot indicates the number of discriminant proteins in within that term. (**b**) *Kyoto Encyclopedia of Genes and Genomes* (KEGG) pathway enrichment analysis is represented as a diagram showcasing the higher and lower abundance of discriminant proteins.

The detailed protein analysis revealed that a total of 1483 and 1544 proteins were significantly enriched in the heart samples of the thyme and oregano groups, respectively. These proteins spanned three major GO categories: biological processes, molecular function, and cellular component. The most important enriched GO terms and higher and lower relative abundance of discriminant proteins are represented in Figure 4a and Table 1, respectively.

Regarding the lower abundance of discriminant proteins, numerous enriched GO terms indicated a mitochondrial involvement. Over-representation of GO biological processes, molecular functions, and cellular components associated with mitochondrial function was observed following the essential oil treatments, particularly at both doses of thyme. The most enriched biological processes were related to the mitochondrial protein translation (GO:0070126, GO:007125, GO:0032543) and muscle contraction (GO:0030049; GO:0006936). Notably, a significant proportion of the enriched cellular components were closely related to mitochondria and the mitochondrial respiratory chain (GO:0005743, GO:0005750, GO:0005758) as well as the actin cytoskeleton (GO:0015269). Similarly, higher abundance of discriminant proteins related to biological processes linked to mitochondria, particularly those involved in negative regulation of respiration (GO:1901856, GO:1903427), were identified. Enrichment of biological processes related to the regulation of calcium transport (GO:0051279) was also observed. As expected, enriched cellular components were associated with the mitochondrial membrane, respiratory chain complexes (GO:0005758, GO:0005743, GO:0045271), and the myosin complex (GO:0005859) (Figure 4a).

Subsequently, we performed a KEGG analysis to investigate the key pathways involved following the administration of essential oils. Notably, the pathway associated with cardiac muscle contraction exhibited significant enrichment among lower abundant discriminant proteins in the thyme-10, thyme-20, and oregano-10 groups. However, we found that this pathway was associated with the enrichment of higher abundant discriminant proteins in the oregano-20 group (Figure 4b). Furthermore, the higher abundant discriminant proteins induced by thyme essential oil exhibited an over-representation of pathways related to Parkinson’s disease and neurological disorders, which are frequently associated with heart failure. The abundance of all significantly enriched proteins found in the heart from the thyme and oregano groups is represented in Table S3.

### 3.4. Decline of Protein Oxidation and Glycoxidation in Plasma

**Figure 5:**
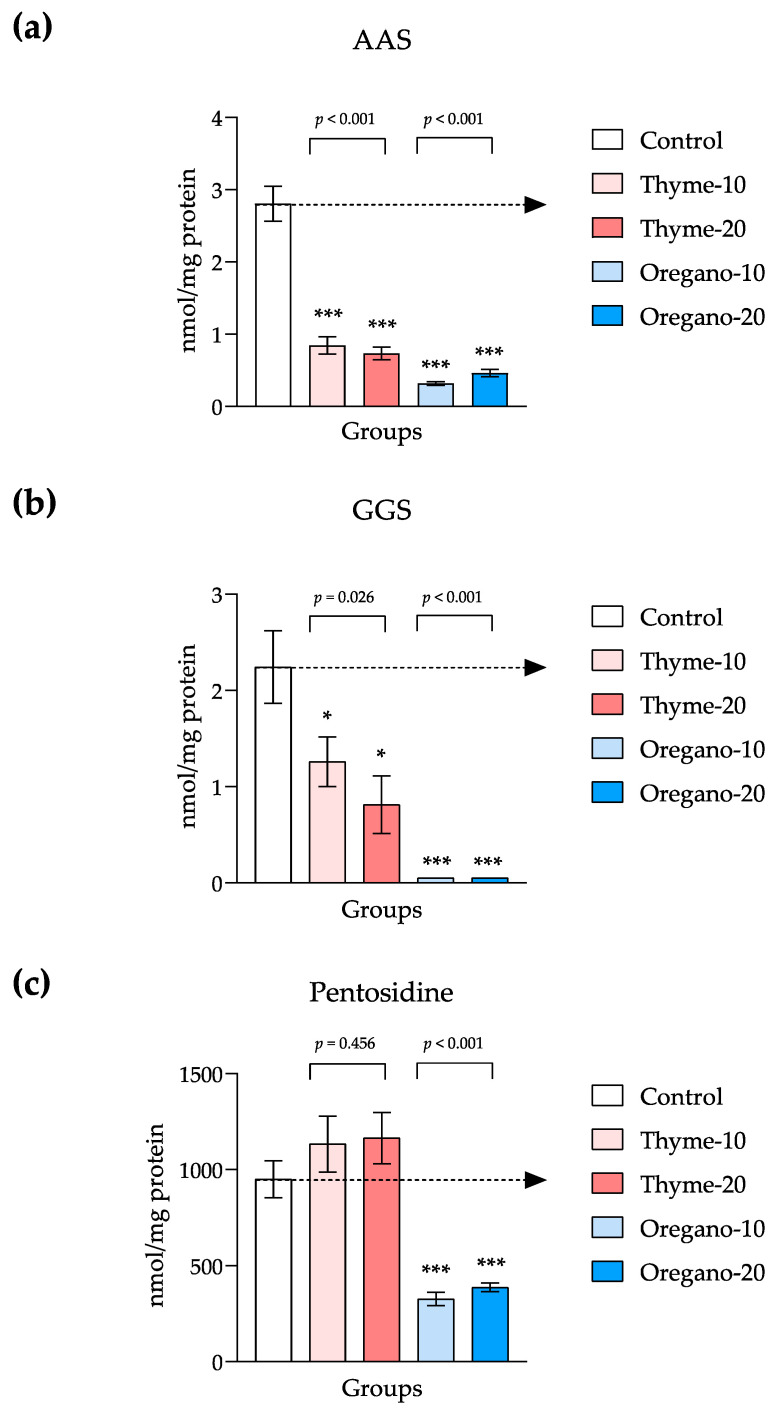
Plasma levels of protein carbonyls and pentosidine in mice from the treatment groups. (**a**) Aminoadipic semialdehyde (AAS); (**b**) Glutamic semialdehyde (GGS); (**c**) Pentosidine levels. The bars represent means ± SEM of nmol/mg. Data were analyzed using one-way ANOVA or the Kruskal–Wallis test, followed by Dunn’s multiple comparison test as post hoc test. (*) *p* < 0.05 and (***) *p* < 0.001 indicate significant differences as compared to the control group. The black arrow indicates control levels.

To validate the observed antioxidant effect detected in the proteomic analysis following the administration of essential oils, we quantified protein carbonyls and pentosidine levels in the plasma of mice from all the experimental groups (Figure 5).

We observed significant main effects on AAS and GGS levels for both thyme- and oregano-derived essential oil treatments. Specifically, one-way ANOVA revealed a significant main effect for the thyme-derived essential oil treatment on plasma levels of AAS (F = 51.77, and *p* < 0.001), and post hoc comparisons showed significant decreases in AAS levels in the thyme-10 and thyme-20 groups as compared to the control group (*p* < 0.001 for both comparisons). Similarly, a significant main effect for the oregano-derived essential oil treatment was also observed (F = 97.76 and *p* < 0.001), and significant decreases in AAS levels were observed in the oregano-10 and oregano-20 groups when compared to the control group (*p* < 0.001 for both comparisons) (Figure 5a). With regard to GGS, one-way ANOVA indicated a significant main effect for the thyme-derived essential oil treatment (F = 5.16, and *p* = 0.026). Post hoc comparisons displayed significant decreases in GGS levels in the thyme-10 and thyme-20 groups when compared to the control group (*p* < 0.05 for both comparisons). In addition, GGS levels were significantly affected by the oregano-derived essential oil treatment using the Kruskal–Wallis rank-sum test (KW = 14.35 and *p* < 0.001), and there were significant decreases in the oregano-10 and oregano-20 groups as compared to the control group (*p* < 0.001 for both comparisons) (Figure 5b).

One-way ANOVA indicated a significant main effect for the oregano-derived essential oil treatment on pentosidine levels (F = 29.25 and *p* < 0.001). Thus, subsequent comparisons displayed significant decreases in pentosidine levels in the oregano-10 and oregano-20 groups as compared to the control group (*p* < 0.001 for both comparisons). Unlike oregano, pentosidine was not affected by the thyme-derived essential oil treatment (Figure 5c).

### 3.5. Decreased Levels in TMAO and Cholesterol in Plasma as Indicators of Cardiovascular Health

**Figure 6:**
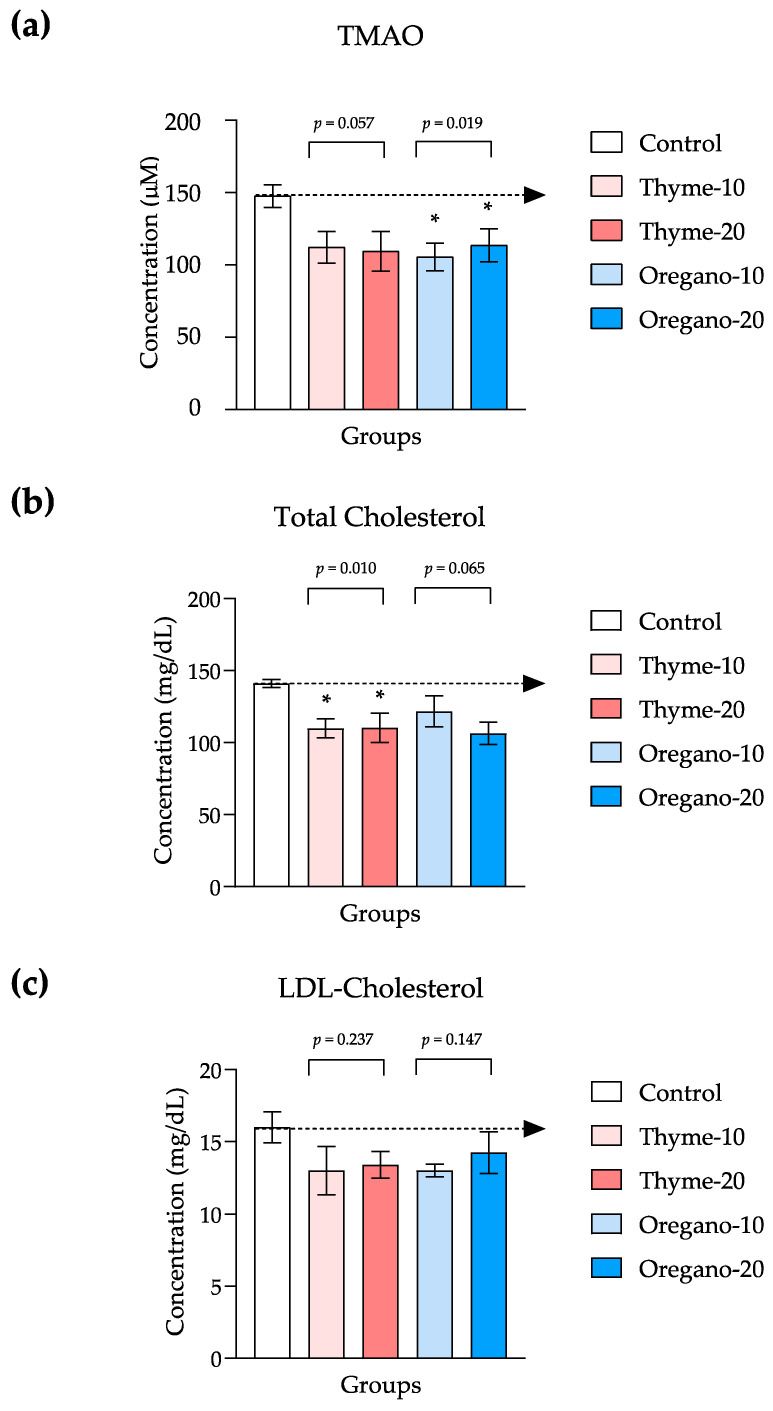
Plasma levels of trimethylamine N-oxide (TMAO) and cholesterol in mice from the treatment groups. (**a**) TMAO; (**b**) Total cholesterol; (**c**) LDL cholesterol levels. The bars represent means ± SEM of µM or mg/dL. Data were analyzed using one-way ANOVA or the Kruskal–Wallis test, followed by Dunn’s multiple comparison test as post hoc test. (*) *p* < 0.05 indicates significant differences compared to the control group. The black arrow indicates control levels.

In a complementary approach, we quantified TMAO and cholesterol (total and LDL cholesterol) levels to investigate in the plasma additional markers associated with cardiovascular health and metabolic status in these mice following the administration of thyme- and oregano-derived essential oils (Figure 6).

Overall, we observed reduced TMAO levels for both essential oil treatments. However, one-way ANOVA revealed a significant main effect only for the oregano-derived essential oil treatment (F = 5.59 and *p* = 0.019), while no statistical significance was observed for the thyme-derived essential oil (F = 3.76 and *p* = 0.057). Post hoc comparisons indicated significant decreases in TMAO levels in the oregano-10 and oregano-20 groups as compared to the control group (*p* < 0.05 for both comparisons) (Figure 6a).

With regard to cholesterol, significant differences were evident in total cholesterol levels based on treatment (Figure 6b), although no statistical differences were identified for LDL cholesterol using the Kruskal–Wallis test for both essential oil treatments (Figure 6c). More specifically, one-way ANOVA demonstrated a significant main effect for the thyme-derived essential oil treatment (F = 7.64 and *p* = 0.010), while no such effect was found for the oregano-derived essential oil (F = 3.35 and *p* = 0.065). In this case, comparisons displayed significant decreases in total cholesterol levels in the thyme-10 and thyme-20 groups as compared to the control group (*p* < 0.05 for both comparisons) (Figure 6b).

## 4. Discussion

Gut microbiota dysbiosis is known to play a crucial role in the development and progression of metabolic and cardiovascular diseases [34,35]. Diet significantly influences both the composition and metabolic activity of the gut microbiome [6]. Specifically, the Mediterranean diet has been strongly linked to rebalancing populations of gut microbiota and the metabolites they generate [36], which directly enhances cardiovascular health [37,38]. In our prior study, we found prebiotic effects of essential oils extracted from savory, parsley, and rosemary on gut microbiota, resulting in an increase in commensal bacteria [15].

In this study, we applied a multi-omics approach using a humanized murine model that harbored gut microbiota obtained from patients with CAD and T2DM. Our aim was to explore the effects of the thyme- and oregano-derived essential oils administered as nutraceuticals for managing a complex cardiometabolic condition. We observed alterations in gut microbiota composition following the administration of essential oils, including increased levels of the *Lactobacillus* genus. Concurrently with the heightened presence of beneficial microbiota populations in feces, we observed favorable changes in metabolites associated with gut microbiota. Specifically, elevated levels of SCFAs in the fecal samples were accompanied by an overall reduction in TMAO levels in the plasma, a pro-atherogenic substance that is derived from a precursor produced by gut microbiota. Moreover, proteomic analyses of cardiac tissues revealed that the primary mechanisms impacted due to the essential oil treatments were linked to oxidation, mitochondrial function, and cardiac contraction. Ultimately, the antioxidant profile induced by the essential oils was substantiated through the reduction in plasma levels of carbonyls and pentosidine, along with an amelioration of cardiovascular health risk factors, such as cholesterol.

Previous studies have indicated that dietary supplementation with a blend of carvacrol and thymol, both active ingredients found in oregano, reduces weaning-induced intestinal oxidative stress. This supplementation leads to a decrease in populations of the *Enterococcus* genus and *E. coli*, while increasing the population of the *Lactobacillus* genus in the jejunum [39]. In the case of thyme, active components, such as eucalyptol and linalool, possess potent antioxidant and antimicrobial properties that can influence the intestinal microbiota of mice, enhancing the relative abundance of *Lactobacillus* [40,41]. Regarding the gut microbiota, we analyzed both alpha and beta diversity to study the intra- and inter-group species composition, respectively. The results revealed an overall increase in total species diversity following the oral administration of both essential oils. Of particular significance, the administration of thyme- and oregano-derived essential oils led to an augmented abundance of the *Lactobacillus* genus in mice. This observation is consistent with earlier studies involving patients with diabetes, where a reduction in *Lactobacillus* and a potential increase in other harmful bacterial populations were reported [42]. The *Lactobacillus* genus consists of beneficial commensal bacteria, and multiple studies have demonstrated their expansion following the essential oil treatments. This increase is often accompanied by a decline in other bacterial populations that do not confer benefits [10,15]. In line with this, we also noted a decreased abundance of the *Colidextribacter* genus in mice treated with essential oils, particularly among those receiving the highest dose of oregano-derived essential oil. Notably, the presence of this genus is considered indicative of obesity and T2DM [43].

Gut microbiota activity is closely related to SCFA production, whose high levels exert a positive impact on the host metabolism [11]. As we detected alterations in microbiota composition subsequent to the administration of these essential oils, we further explored their influence on SCFA levels in fecal samples. We observed heightened levels of acetic, propionic, and butyric acids, which have been linked to augmented mucus production and increased expression of tight junctions. Such effects contribute to the enhancement of gut barrier integrity, alongside a reduction in insulin secretion [44]. Our finding of a significant increase in the abundance of the *Lactobacillus* genus through the use of thyme- and oregano-derived essential oils aligns with a previous study wherein we reported an increase in the abundance of this genus and acetic acid within a similar humanized model [15]. Conversely, we noted a decrease in the abundance of the *Colidextribacter* genus after the oregano-derived essential oil treatment. This finding is congruent with prior studies suggesting an inverse correlation between its microbial activity and the levels of acetic and butyric acids [45]. This correlation is consistent with our results, as the oregano-derived essential oil treatment resulted in a reduction in the abundance of the *Colidextribacter* genus, likely contributing to the observed increase in acetic and butyric acids in the animals treated with this essential oil. Furthermore, we observed an overall reduction in TMAO levels following the administration of both essential oils. Specific microbial populations use L-carnitine as a precursor for the synthesis of TMA, which undergoes rapid absorption into the portal circulation through passive diffusion across enterocyte membranes. Subsequently, hepatic flavin-containing monooxygenases catalyze its conversion to TMAO. Considering the established link between TMAO and cardiovascular risk, this decline substantiates the beneficial impact of these essential oils and aligns with the effects observed in other essential oils derived from parsley and rosemary [15].

Given that our humanized model contained microbiota from patients with severe CAD, we investigated the protein profile in cardiac tissue. The aim was to evaluate the mechanisms triggered by these essential oils and elucidate the tissue-specific molecular pathways affected by the treatments. Our findings revealed that the essential oil treatments led to significant alterations in lower abundance discriminant proteins associated with mitochondrial function, oxidation, and cardiac contraction. For instance, following the oregano-derived essential oil treatment, we noted a decrease in the abundance of small ubiquitin-like modifier-3 (SUMO3), a protein involved in a process that modifies the function of other proteins (i.e., SUMOylation) [46]. SUMOylation is a reversible post-translational modification that influences protein activity, stability, interaction, and localization [47]. This regulatory process plays a pivotal role in cardiac conditions and mitochondrial dynamics amid pronounced oxidative stress [47,48,49]. Furthermore, we identified over-represented pathways linked to cardiac contraction. Specifically, the results revealed lower representation of LIM domain binding 3 (LDB3) and tropomyosin proteins after the administration of these essential oils. LDB3 is a protein stabilizing the sarcomere during contraction in cardiac and skeletal muscles, and mutations in this protein have been linked to severe cardiomyopathies [50]. Tropomyosin, a member of the actin regulatory protein family, holds a central role in calcium-dependent cardiac muscle contraction [51,52]. These observations mirror previous studies emphasizing the capacity of natural products to modulate mitochondrial activity in cardiac disease contexts [53]. A reduced cardiac rate could imply decreased mitochondrial activity due to lowered energy demand. Consequently, it was unsurprising to identify enriched molecular functions pertaining to mitochondrial function. As a result of diminished mitochondrial activity, cardiac cells could generate fewer ROS levels, undesirable byproducts of the mitochondrial oxidative phosphorylation process. This reduced energy demand could correspondingly mitigate oxidative stress. The observed increase in the abundance of *Lactobacillus* following the essential oil treatments also aligns with the potential antioxidant effect elucidated by our proteomic analyses. Research suggests certain *Lactobacillus* species possess the capacity to reduce free radical levels by altering redox state, lipid peroxidation, and superoxide dismutase activity [44]. Furthermore, we found a decrease in the abundance of the *Colidextribacter* genus in fecal samples, consistent with prior studies implying an antioxidant and hypoglycemic impact attributed to *Colidextribacter* [54]. This aligns with our previous findings, as we documented antioxidant effects of essential oils derived from savory, parsley, and rosemary, that were associated with the modulation of commensal microbiota [15].

To further investigate the antioxidant profile induced by thyme- and oregano-derived essential oil treatments, we quantified levels of protein carbonyls (i.e., AAG and GGS) and pentosidine in the plasma of mice, serving as indicators of oxidation and glycoxidation, respectively. This interest is motivated by the pro-oxidative milieu prevalent in cardiometabolic dysfunction, characterized by an increase in (glyco)oxidized proteins as a consequence of ROS-mediated damage. Additionally, levels of protein carbonyls in the tissues of individuals with T2DM are elevated due to the presence of circulating reducing sugars and their subsequent metabolic byproducts (glyoxal/methylglyoxal), which induce carbonylation of human serum proteins [18]. Our findings demonstrated a notable reduction in AAS and GGS levels following the administration of both thyme- and oregano-derived essential oils, alongside a decrease in pentosidine levels following the administration of oregano-derived essential oil.

In accordance with the observed decline in protein carbonylation in treated mice, we noted a reduction in the abundance of proteins involved in carbonyl metabolism subsequent to the essential oil treatments. Remarkably, the aldose reductase-related protein 2 exhibited reduced levels after the thyme-derived essential oil treatment, an enzyme known to be implicated in the pathogenesis of diabetic complications [55]. Furthermore, alcohol dehydrogenase [NADP (+)], responsible for catalyzing NADPH-dependent reductions in carbonyls into their corresponding alcohols [56], and the mitochondrial delta-1-pyrroline-5-carboxylate dehydrogenase, responsible for facilitating the second step of the proline degradation pathway [57], manifested decreased abundances subsequent to the administration of the oregano-derived essential oil.

These findings are congruent with the diminished levels of proteins associated with mitochondrial function, as deduced from the functional enrichment analysis. Notably, a decrease in proteins linked to mitochondrial SUMOylation was evident, aligning with the decreased concentration of protein carbonyls and pentosidine. This connection can be elucidated by the direct influence of oxidative stress and cellular ROS-mediated detriment [58]. Consequently, it is plausible to posit that the protection against ROS generation and (glyco)oxidative stress in mice treated with essential oils is manifested through the reduced plasma carbonylation. It is pertinent to underscore that protein carbonylation plays a well-established role in the molecular pathogenesis of T2DM [59]. Plasma protein carbonyls function as dependable markers of obesity induced by T2DM [60], and the diminished concentrations of these entities in the plasma of T2DM patients hold promising prospects for therapeutics and diagnostics in humans [61].

The alterations in gut microbial abundances induced by the administration of both essential oils, as previously detailed, can exert an impact on the oxidative status. Notably, *Lactobacillus* demonstrated a negative correlation with intestinal oxidative stress in animals due to its capacity to inhibit ROS production through colon digesta fermentation [62,63]. It is noteworthy that patients with diabetes often exhibit diminished levels of the *Bifidobacterium* and *Lactobacillus* genera, leading to elevated levels of the *Bacteroides*, *Prevotella*, *Peptococcus*, *Clostridium*, *Proteus*, *Staphylococcus*, and *Candida* genera [42]. Furthermore, individuals with T2DM tend to possess reduced populations of butyrate-producing bacteria [42]. Furthermore, the diminished levels of TMAO following the administration of thyme- and oregano-derived essential oils can be attributed to alterations in the gut microbial populations and likely to the reduction in the oxidative activity of hepatic monooxygenases due to antioxidant effects. These potential mechanisms for TMAO reduction have also been considered in previous research involving other essential oils [15].

Hence, the beneficial effects of both essential oils likely stem from a multitude of factors. These encompass heightened mitochondrial activity, decreased oxidative markers due to the scavenging capabilities of essential oil constituents, and the restoration of microbiota equilibrium. Collectively, these factors contribute to the amelioration of ROS levels, the stimulation of enhanced SCFA production and the mitigation of risk factors for cardiovascular health. Our findings affirm the prebiotic and antioxidant attributes inherent in essential oils sourced from commonly used Mediterranean diet herbs, such as thyme and oregano. These observations align with our earlier investigations involving essential oils derived from different herbs [15]. Moreover, the apparent attenuation of mitochondrial activity resulting from the essential oil treatments correlates with the pronounced reduction in oxidative markers identified within the treated animals.

## 5. Conclusions

Our study offers innovative insights into the exploration of the role of gut microbiota in health and disease. The Mediterranean diet, rich in plant-derived compounds, has exhibited a favorable influence on gut microbiota composition and their metabolites. Our investigation has revealed that the inclusion of essential oils extracted from thyme and oregano in the diet imparts prebiotic and antioxidant benefits. Thus, we infer that the dietary integration of these essential oils as nutraceuticals could potentially exert a substantial protective effect against cardiometabolic disorders. However, to gain a comprehensive understanding of the underlying mechanisms and to substantiate these findings in human subjects, further studies are imperative.

## Figures and Tables

**Table 1:** Abundance of significant proteins related to oxidation and cardiac contraction found in heart samples of the thyme and oregano groups. The GeneCards database (http://www.genecards.org/) (accessed on 10 April 2023) was used to identify the biological function of the proteins.

Protein Name	Gene Name	*p*-Value	Fold-Change	Biological Function	Accession Number	Treatment/Dose
Aldose reductase-related protein 2	AKR1B8	0.03	−0.98	Carbonyl metabolism	P45377	Thyme/10
Isoform 2 of Aspartyl/asparaginyl beta-hydroxylase	ASPH	<0.0001 <0.0001	−0.99 −1.11	Cardiac contraction	Q8BSY0	Thyme/10 Thyme/20
Tropomyosin beta chain	TPM2	0.01 0.006	−0.79 −0.87	Cardiac contraction	P58774	Thyme/10 Thyme/20
Isoform 5 of LIM domain-binding protein 3	LDB3	<0.0001	−1.81	Cardiac contraction	Q9JKS4	Oregano/10
Small ubiquitin-related modifier 3	SUMO3	0.003 0.002	−1.49 −1.64	SUMOylation, process regulated by cellular oxidative stress	Q9Z172	Oregano/10 Oregano/20
NADH-ubiquinone oxidoreductase chain 4	MTND4	0.04	−1.34	Mitochondrial respiratory chain	P03911	Oregano/20
Delta-1-pyrroline-5-carboxylate dehydrogenase, mitochondrial	ALDH4A1	0.03	−0.89	Carbonyl metabolism	Q8CHT0	Oregano/10
Alcohol dehydrogenase [NADP (+)]	AKR1A1	0.03	−0.61	Carbonyl metabolism	Q9JII6	Oregano/10

## Data Availability

Data are presented as mean or median of raw data in this study. Raw data are available on request from the corresponding authors.

## Supplementary Materials

The following supporting information can be downloaded at https://www.mdpi.com/article/10.3390/antiox12081643/s1, Table S1: Proximate composition and energy content of the diet used for mice; Table S2: Composition of essential oils derived from thyme and oregano using SPME-GC-MS; Table S3: Abundance of significant proteins in heart samples of the thyme and oregano groups.

## References

[1] Saljoughian S., Roohinejad S., Bekhit A.E.D.A., Greiner R., Omidizadeh A., Nikmaram N., Mousavi Khaneghah A. (2018). The Effects of Food Essential Oils on Cardiovascular Diseases: A Review. Crit. Rev. Food Sci. Nutr..

[2] Lacroix S., Cantin J., Nigam A. (2017). Contemporary Issues Regarding Nutrition in Cardiovascular Rehabilitation. Ann. Phys. Rehabil. Med..

[3] Dos Reis Padilha G., Sanches Machado d’Almeida K., Ronchi Spillere S., Corrêa Souza G. (2018). Dietary Patterns in Secondary Prevention of Heart Failure: A Systematic Review. Nutrients.

[4] Yap P.S.X., Yiap B.C., Ping H.C., Lim S.H.E. (2014). Essential Oils, A New Horizon in Combating Bacterial Antibiotic Resistance. Open Microbiol. J..

[5] Bentham Science Publisher B.S.P. (2006). The Gut Microbiota and Lipid Metabolism: Implications for Human Health and Coronary Heart Disease. Curr. Med. Chem..

[6] Mayneris-Perxachs J., Castells-Nobau A., Arnoriaga-Rodríguez M., Martin M., de la Vega-Correa L., Zapata C., Burokas A., Blasco G., Coll C., Escrichs A. (2022). Microbiota Alterations in Proline Metabolism Impact Depression. Cell Metab..

[7] Hashemipour H., Kermanshahi H., Golian A., Veldkamp T. (2013). Metabolism and Nutrition: Effect of Thymol and Carvacrol Feed Supplementation on Performance, Antioxidant Enzyme Activities, Fatty Acid Composition, Digestive Enzyme Activities, and Immune Response in Broiler Chickens. Poult. Sci..

[8] Alagawany M. (2015). Biological Effects and Modes of Action of Carvacrol in Animal and Poultry Production and Health—A Review. Adv. Anim. Vet. Sci..

[9] Hrncir T., Hrncirova L., Kverka M., Hromadka R., Machova V., Trckova E., Kostovcikova K., Kralickova P., Krejsek J., Tlaskalova-Hogenova H. (2021). Gut Microbiota and NAFLD: Pathogenetic Mechanisms, Microbiota Signatures, and Therapeutic Interventions. Microorganisms.

[10] Li S.Y., Ru Y.J., Liu M., Xu B., Péron A., Shi X.G. (2012). The Effect of Essential Oils on Performance, Immunity and Gut Microbial Population in Weaner Pigs. Livest. Sci..

[11] Miller T.L., Wolin M.J. (1996). Pathways of Acetate, Propionate, and Butyrate Formation by the Human Fecal Microbial Flora. Appl. Environ. Microbiol..

[12] Macfarlane S., Macfarlane G.T. (2003). Regulation of Short-Chain Fatty Acid Production. Proc. Nutr. Soc..

[13] Luu M., Pautz S., Kohl V., Singh R., Romero R., Lucas S., Hofmann J., Raifer H., Vachharajani N., Carrascosa L.C. (2019). The Short-Chain Fatty Acid Pentanoate Suppresses Autoimmunity by Modulating the Metabolic-Epigenetic Crosstalk in Lymphocytes. Nat. Commun..

[14] Fernandes J., Su W., Rahat-Rozenbloom S., Wolever T.M.S., Comelli E.M. (2014). Adiposity, Gut Microbiota and Faecal Short Chain Fatty Acids Are Linked in Adult Humans. Nutr. Diabetes.

[15] Sánchez-Quintero M.J., Delgado J., Medina-Vera D., Becerra-Muñoz V.M., Queipo-Ortuño M.I., Estévez M., Plaza-Andrades I., Rodríguez-Capitán J., Sánchez P.L., Crespo-Leiro M.G. (2022). Beneficial Effects of Essential Oils from the Mediterranean Diet on Gut Microbiota and Their Metabolites in Ischemic Heart Disease and Type-2 Diabetes Mellitus. Nutrients.

[16] Ray P.D., Huang B.W., Tsuji Y. (2012). Reactive oxygen species (ROS) homeostasis and redox regulation in cellular signaling. Cell Signal..

[17] Bețiu A.M., Noveanu L., Hâncu I.M., Lascu A., Petrescu L., Maack C., Elmér E., Muntean D.M. (2022). Mitochondrial Effects of Common Cardiovascular Medications: The Good, the Bad and the Mixed. Int. J. Mol. Sci..

[18] Sun Y., Lu Y., Saredy J., Wang X., Drummer Iv C., Shao Y., Saaoud F., Xu K., Liu M., Yang W.Y. (2020). ROS systems are a new integrated network for sensing homeostasis and alarming stresses in organelle metabolic processes. Redox Biol..

[19] Luna C., Arjona A., Dueñas C., Estevez M. (2021). Allysine and α-Aminoadipic Acid as Markers of the Glyco-Oxidative Damage to Human Serum Albumin under Pathological Glucose Concentrations. Antioxidants.

[20] Sell D.R., Strauch C.M., Shen W., Monnier V.M. (2007). 2-Aminoadipic Acid Is a Marker of Protein Carbonyl Oxidation in the Aging Human Skin: Effects of Diabetes, Renal Failure and Sepsis. Biochem. J..

[21] Velichkova S., Foubert K., Pieters L. (2021). Natural Products as a Source of Inspiration for Novel Inhibitors of Advanced Glycation Endproducts (AGEs) Formation. Planta Medica.

[22] Matera R., Lucchi E., Valgimigli L. (2023). Plant Essential Oils as Healthy Functional Ingredients of Nutraceuticals and Diet Supplements: A Review. Molecules.

[23] Le Bastard Q., Ward T., Sidiropoulos D., Hillmann B.M., Chun C.L., Sadowsky M.J., Knights D., Montassier E. (2018). Fecal Microbiota Transplantation Reverses Antibiotic and Chemotherapy-Induced Gut Dysbiosis in Mice. Sci. Rep..

[24] Zhang Y., Huang R., Cheng M., Wang L., Chao J., Li J., Zheng P., Xie P., Zhang Z., Yao H. (2019). Gut Microbiota from NLRP3-Deficient Mice Ameliorates Depressive-like Behaviors by Regulating Astrocyte Dysfunction via CircHIPK2. Microbiome.

[25] Ubeda C., Bucci V., Caballero S., Djukovic A., Toussaint N.C., Equinda M., Lipuma L., Ling L., Gobourne A., No D. (2013). Intestinal Microbiota Containing Barnesiella Species Cures Vancomycin-Resistant Enterococcus Faecium Colonization. Infect. Immun..

[26] Reikvam D.H., Erofeev A., Sandvik A., Grcic V., Jahnsen F.L., Gaustad P., McCoy K.D., Macpherson A.J., Meza-Zepeda L.A., Johansen F.E. (2011). Depletion of Murine Intestinal Microbiota: Effects on Gut Mucosa and Epithelial Gene Expression. PLoS ONE.

[27] Kennedy E.A., King K.Y., Baldridge M.T. (2018). Mouse Microbiota Models: Comparing Germ-Free Mice and Antibiotics Treatment as Tools for Modifying Gut Bacteria. Front. Physiol..

[28] Sanchez-Alcoholado L., Castellano-Castillo D., Jordán-Martínez L., Moreno-Indias I., Cardila-Cruz P., Elena D., Muñoz-Garcia A.J., Queipo-Ortuño M.I., Jimenez-Navarro M. (2017). Role of Gut Microbiota on Cardio-Metabolic Parameters and Immunity in Coronary Artery Disease Patients with and without Type-2 Diabetes Mellitus. Front. Microbiol..

[29] Wu W.K., Panyod S., Ho C.T., Kuo C.H., Wu M.S., Sheen L.Y. (2015). Dietary Allicin Reduces Transformation of L-Carnitine to TMAO through Impact on Gut Microbiota. J. Funct. Foods.

[30] Chen E.Y., Tan C.M., Kou Y., Duan Q., Wang Z., Meirelles G.V., Clark N.R., Ma’ayan A. (2013). Enrichr: Interactive and Collaborative HTML5 Gene List Enrichment Analysis Tool. BMC Bioinform..

[31] Kuleshov M.V., Jones M.R., Rouillard A.D., Fernandez N.F., Duan Q., Wang Z., Koplev S., Jenkins S.L., Jagodnik K.M., Lachmann A. (2016). Enrichr: A Comprehensive Gene Set Enrichment Analysis Web Server 2016 Update. Nucleic Acids Res..

[32] Xie Z., Bailey A., Kuleshov M.V., Clarke D.J.B., Evangelista J.E., Jenkins S.L., Lachmann A., Wojciechowicz M.L., Kropiwnicki E., Jagodnik K.M. (2021). Gene Set Knowledge Discovery with Enrichr. Curr. Protoc..

[33] Utrera M., Morcuende D., Rodríguez-Carpena J.G., Estévez M. (2011). Fluorescent HPLC for the Detection of Specific Protein Oxidation Carbonyls—α-Aminoadipic and γ-Glutamic Semialdehydes—In Meat Systems. Meat Sci..

[34] Shen Y., Wang P., Yang X., Chen M., Dong Y., Li J. (2023). Untargeted Metabolomics Unravel Serum Metabolic Alterations in Smokers with Hypertension. Front. Physiol..

[35] Tilg H., Kaser A. (2011). Gut Microbiome, Obesity, and Metabolic Dysfunction. J. Clin. Investig..

[36] Garcia-mantrana I., Selma-royo M., Alcantara C., Collado M.C. (2018). Shifts on Gut Microbiota Associated to Mediterranean Diet Adherence and Specific Dietary Intakes on General Adult Population. Front. Microbiol..

[37] Liang K.W., Lee C.L., Liu W.J. (2022). Lower All-Cause Mortality for Coronary Heart or Stroke Patients Who Adhere Better to Mediterranean Diet-An. Nutrients.

[38] Estruch R., Ros E., Salvadó J.S., Covas M., Corella D., Arós F., Gracia E.G., Gutiérrez V.R., Fiol M., Lapetra J. (2018). Primary Prevention of Cardiovascular Disease with a Mediterranean Diet Supplemented with Extra-Virgin Olive Oil or Nuts. N. Engl. J. Med..

[39] Wei H.K., Xue H.X., Zhou Z.X., Peng J. (2017). A Carvacrol-Thymol Blend Decreased Intestinal Oxidative Stress and Influenced Selected Microbes without Changing the Messenger RNA Levels of Tight Junction Proteins in Jejunal Mucosa of Weaning Piglets. Animal.

[40] Scherer R., Junior S.B., de Albuquerque R., Godoy H.T. (2014). Microencapsulated Eucalyptol and Eugenol as Growth Promoters in Broilers. Braz. J. Food Res..

[41] Wang L., Zhang Y., Fan G., Ren J.N., Zhang L.L., Pan S.Y. (2019). Effects of Orange Essential Oil on Intestinal Microflora in Mice. J. Sci. Food Agric..

[42] Tilg H., Moschen A.R. (2014). Microbiota and Diabetes: An Evolving Relationship. Gut.

[43] Yu Z., Yu X.F., Kerem G., Ren P.G. (2022). Perturbation on Gut Microbiota Impedes the Onset of Obesity in High Fat Diet-Induced Mice. Front. Endocrinol..

[44] Zununi Vahed S., Barzegari A., Zuluaga M., Letourneur D., Pavon-Djavid G. (2018). Myocardial Infarction and Gut Microbiota: An Incidental Connection. Pharmacol. Res..

[45] Xia J., Lv L., Liu B., Wang S., Zhang S., Wu Z., Yang L., Bian X., Wang Q., Wang K. (2022). Akkermansia Muciniphila Ameliorates Acetaminophen-Induced Liver Injury by Regulating Gut Microbial Composition and Metabolism. Microbiol. Spectr..

[46] Hotz P.W., Müller S., Mendler L. (2021). SUMO-Speci Fi c Isopeptidases Tuning Cardiac SUMOylation in Health and Disease. Front. Mol. Biosci..

[47] Pober J., Min W. (2023). SRF SUMOylation Modulates Smooth Muscle Phenotypic Switch and Vascular Remodeling.

[48] Xiu D., Wang Z., Cui L., Jiang J., Yang H., Liu G. (2018). Sumoylation of SMAD 4 Ameliorates the Oxidative Stress-Induced Apoptosis in Osteoblasts. Cytokine.

[49] Stankovic-valentin N., Melchior F. (2018). Molecular Aspects of Medicine Control of SUMO and Ubiquitin by ROS: Signaling and Disease Implications. Mol. Asp. Med..

[50] Savarese M., Sarparanta J., Vihola A. (2020). Panorama of the Distal Myopathies. Acta Myol..

[51] Pavadai E., Rynkiewicz M.J., Yang Z., Gould I.R. (2022). Modulation of Cardiac Thin Filament Structure by Phosphorylated Troponin-I Analyzed by Protein-Protein Docking and Molecular Dynamics Simulation. Arch. Biochem. Biophys..

[52] Chalovich J.M., Zhu L., Johnson D., Irving T.C. (2022). Hypertrophic Cardiomyopathy Mutations of Troponin Reveal Details of Striated Muscle Regulation. Front. Physiol..

[53] Boeing T., Reis Lívero F.A.D., de Souza P., de Almeida D.A.T., Donadel G., Lourenço E.L.B., Gasparotto A. (2023). Natural Products as Modulators of Mitochondrial Dysfunctions Associated with Cardiovascular Diseases: Advances and Opportunities. J. Med. Food.

[54] Jiang X., Sun B., Zhou Z. (2022). Preclinical Studies of Natural Products Targeting the Gut Microbiota: Beneficial Effects on Diabetes. J. Agric. Food Chem..

[55] Lau E.T., Cao D., Lin C., Chung S.K., Chung S.S. (1995). Tissue-Specific Expression of Two Aldose Reductase-like Genes in Mice: Abundant Expression of Mouse Vas Deferens Protein and Fibroblast Growth Factor-Regulated Protein in the Adrenal Gland. Biochem. J..

[56] Penning T.M., Palackal N.T., Lee S.H., Blair I., Yu D., Berlin J.A., Field J.M., Harvey R.G. (2003). Aldo-Keto Reductases and the Metabolic Activation of Polycyclic Aromatic Hydrocarbons. ACS Symp. Ser..

[57] Bogner A.N., Stiers K.M., Tanner J.J. (2021). Structure, Biochemistry, and Gene Expression Patterns of the Proline Biosynthetic Enzyme Pyrroline-5-Carboxylate Reductase (PYCR), an Emerging Cancer Therapy Target. Amino Acids.

[58] Estévez M., Díaz-Velasco S., Martínez R. (2022). Protein Carbonylation in Food and Nutrition: A Concise Update. Amino Acids.

[59] Hecker M., Wagner A.H. (2018). Role of Protein Carbonylation in Diabetes. J. Inherit. Metab. Dis..

[60] Bollineni R.C., Fedorova M., Blüher M., Hoffmann R. (2014). Carbonylated Plasma Proteins as Potential Biomarkers of Obesity Induced Type 2 Diabetes Mellitus. J. Proteome Res..

[61] Almogbel E., Rasheed N. (2019). Elevated Levels of Protein Carbonylation in Patients with Diabetic Nephropathy: Therapeutic and Diagnostic Prospects. Am. J. Med. Sci..

[62] Xu C.C., Yang S.F., Zhu L.H., Cai X., Sheng Y.S., Zhu S.W., Xu J.X. (2014). Regulation of N-Acetyl Cysteine on Gut Redox Status and Major Microbiota in Weaned Piglets. J. Anim. Sci..

[63] Sun J., Hu X.L., Le G.W., Shi Y.H. (2010). Lactobacilli Prevent Hydroxy Radical Production and Inhibit Escherichia Coli and Enterococcus Growth in System Mimicking Colon Fermentation. Lett. Appl. Microbiol..

