# Supplementary material for: Multi-Omics Approach Reveals Prebiotic and Potential Antioxidant Effects of Essential Oils from the Mediterranean Diet on Cardiometabolic Disorder Using Humanized Gnotobiotic Mice
Source: Antioxidants (Basel). 2023 Aug 20;12(8):1643. doi: 10.3390/antiox12081643 (PMC10451832; doi:10.3390/antiox12081643)
Supplement: Supplementary file 1 [file antioxidants-12-01643-s001.zip › antioxidants-2541348-supplementary.pdf]

Table S1. Proximate composition and energy content of the diet used for mice.

Crude Nutrient	(%)	Additive	Per kg
Crude Protein	22.0	Vitamin A [IU]	15,000
Crude fat	23.6	Vitamin D_3 [IU]	1,500
Crude fiber	5.7	Vitamin E [mg]	150
Crude ash	5.4	Vitamin C [mg]	30
Starch	6.8		
Sugar	21.1	Copper [mg]	12
**Energy: 19.3 MJ [or kcal] ME/kg**			

Table S2. Composition of essential oils derived from thyme and oregano using SPME-GC-MS.

Essential oil derived from thyme

**Compound Name**	**CAS#**	**Match Factor**	**Area**	**Relative abundance %**
Hexanal	66-25-1	97.29	55245	0.055
Cyclotrisiloxane. hexamethyl-	541-05-9	97.81	213814	0.213
Heptanal	111-71-7	98.76	79477	0.079
Benzaldehyde	100-52-7	99.50	811026	0.808
Hexanoic acid	142-62-1	98.00	55096	0.055
Octanal	124-13-0	99.00	281799	0.281
Decane	124-18-5	98.40	255001	0.254
Benzyl alcohol	100-51-6	99.72	12395148	12.349
o-Cymene	527-84-4	95.92	69140	0.069
Eucalyptol	470-82-6	99.50	47898429	47.722
1-Octanol	111-87-5	97.40	40472	0.040
Undecane. 4.7-dimethyl-	17301-32-5	93.76	73999	0.074
1.6-Octadien-3-ol. 3.7-dimethyl- (Linalool)	78-70-6	99.26	23519627	23.433
Undecane. 4.7-dimethyl-	17301-32-5	93.10	53544	0.053
Octanoic acid	124-07-2	97.58	382622	0.381
(+)-2-Bornanone	464-49-3	99.02	476465	0.475
1-Propanone. 1-phenyl-	93-55-0	96.19	36672	0.037
endo-Borneol	507-70-0	97.87	208895	0.208
Terpinen-4-ol	562-74-3	98.05	1979418	1.972
α-Terpineol	98-55-5	98.06	3810973	3.797
Linalyl acetate	115-95-7	98.08	1681086	1.675
2-Decenal. (Z)-	2497-25-8	93.36	186523	0.186
Thymol	89-83-8	97.49	1734983	1.729

Phenol. 2-methyl-5-(1-methylethyl)- (Carvacrol)	499-75-2	98.72	958727	0.955
Isobornyl acetate	125-12-2	96.52	193313	0.193
Glycerol 1.2-diacetate	102-62-5	94.09	88140	0.088
3-Cyclohexene-1-methanol. α. α.4-trimethyl-. acetate (1-α-Terpinyl acetate)	80-26-2	96.80	768268	0.765
2.6-Octadien-1-ol. 3.7-dimethyl-. acetate. (Z)- (Nerol acetate)	141-12-8	96.61	339273	0.338
1-Nonadecene	18435-45-5	96.05	509024	0.507
Caryophyllene	87-44-5	98.97	830160	0.827
Caryophyllene oxide	1139-30-6	94.05	383885	0.382

Essential oil derived from oregano

**Compound Name**	**CAS#**	**Match Factor**	**Area**	**Relative abundance %**
Propylene Glycol	57-55-6	97.80	160791	0.235
Hexanal	66-25-1	96.49	18884	0.028
Heptanal	111-71-7	93.99	36090	0.053
Camphene	79-92-5	98.27	157767	0.231
1-Octen-3-ol	3391-86-4	96.84	105410	0.154
Octanal	124-13-0	98.34	138950	0.203
Decane	124-18-5	98.72	329778	0.482
Benzyl alcohol	100-51-6	98.76	710454	1.038
o-Cymene	527-84-4	98.80	1349311	1.972
Nonane. 2.6-dimethyl-	17302-28-2	94.77	16966	0.025
Eucalyptol	470-82-6	99.45	2522700	3.686
γ-Terpinene	99-85-4	96.45	151752	0.222
1.6-Octadien-3-ol. 3.7-dimethyl-	78-70-6	97.09	1206835	1.763
Octane. 5-ethyl-2-methyl-	62016-18-6	93.56	52184	0.076
**Hexane. 3.3-dimethyl-**	563-16-6	94.34	19494	0.028

**endo-Borneol**	507-70-0	97.09	95727	0.140
**Terpinen-4-ol**	562-74-3	93.25	69744	0.102
**Thymol**	89-83-8	98.82	26238606	38.338
**Phenol. 2-methyl-5-(1-methylethyl)- (Carvacrol)**	499-75-2	98.86	14155626	20.683
**Isobornyl acetate**	125-12-2	99.16	15799968	23.086
**Glycerol 1.2-diacetate**	102-62-5	97.76	850967	1.243
**1-Octadecene**	112-88-9	97.91	670402	0.980
**Caryophyllene**	87-44-5	99.63	3063608	4.476
**Phenol. 2.4-bis(1.1-dimethylethyl)-**	96-76-4	93.25	223094	0.326
Caryophyllene oxide	1139-30-6	94.57	294348	0.430

Table S3. Abundance of significantly proteins in heart samples of the thyme and oregano groups.

Protein Name	Gene Name	p-Value	Fold-Change	Biological Function	Accesion Number	Treatment / dose (mg/ml)
fN(G),N(G)-dimethylarginine dimethylaminohydrolase 1	DDAH1	0.006	-2.04	Metabolism nitric oxide	Q9CWS0	Thyme/10
Stomatin-like protein 2, mitochondrial	STOML2	0.01	-1.84	Biogenesis and activity of mitochondria regulation	Q99JB2	Thyme/10
DNA-(apurinic or apyrimidinic site) lyase	APEX1	0.02	-1.59	Redox regulation of transcriptional factors	P28352	Thyme/10
Hydroxyacylglutathione hydrolase, mitochondrial	HAGH	0.003	-1.38	Detoxification of methylglyoxal	Q99KB8	Thyme/10
Protein MEMO1	MEMO1	0.01	-1.07	NADH oxidase-induced redox signaling	Q91VH6	Thyme/10
Acidic leucine-rich nuclear phosphoprotein 32 family member A	ANP32A	< 0.0001	-1.19	Oxidative stress regulation via ATM	O35381	Thyme/10
Translation factor GUF1, mitochondrial	GUF1	0.01	-1.01	Mitochondrial protein synthesis	Q8C3X4	Thyme/10
Aldose reductase-related protein 2	AKR1B8	0.03	-0.98	Carbonyl metabolism	P45377	Thyme/10
Alpha-methylacyl-CoA racemase	AMACR	0.04	-0.91	Peroxisomal lipid metabolism	O09174	Thyme/10
Monoglyceride lipase	MGLL	0.009	-0.84	Lipid metabolism	O35678	Thyme/10
28S ribosomal protein S36, mitochondrial	MRPS36	< 0.0001	-0.83	Tricarboxylic acid cycle	Q9CQX8	Thyme/10
Ribosome-recycling factor, mitochondrial	MRRF	0.02	-0.68	Mitochondrial translational machinery	Q9D6S7	Thyme/10
Cytochrome c1, heme protein, mitochondrial	CYC1	0.006	-0.66	Mitochondrial respiratory chain	Q9D0M3	Thyme/10
Dihydropteridine reductase	QDPR	0.02	-0.66	Regeneration of tetrahydrobiopterin (BH4)	Q8BVI4	Thyme/10

Phosphatidylglycerophosphatase and protein-tyrosine phosphatase 1	PTPMT1	0.008	-0.65	Biogenesis and activity of mitochondria regulation	Q66GT5	Thyme/10
Protein SCO1 homolog, mitochondrial	SCO1	0.03	-0.57	Maturation of cytochrome c oxidase	Q5SUC9	Thyme/10
LYR motif-containing protein 4	LYRM4	0.006	-0.55	NSF1 stability and activity regulation	Q8K215	Thyme/10
Prosaposin	PSAP	0.01	-0.54	Lipid metabolism	Q61207	Thyme/10
Triosephosphate isomerase	TPI1	0.003	-0.5	Production of methylglyoxal	P17751	Thyme/10
Heat shock protein 75 kDa, mitochondrial	TRAP1	0.03	0.82	Maintaining mitochondrial function and polarization	Q9CQN1	Thyme/10
ADP/ATP translocase 4	SLC25A31	0.04	0.81	Mitochondrial ADP:ATP antiporter and proton transporter	Q3V132	Thyme/10
Transforming protein RhoA	RHOA	0.04	0.69	Oxidative stress response	Q9QUI0	Thyme/10
Chloride intracellular channel protein 1	CLIC1	0.02	0.69	ROS generation by the NAPDH oxidase	Q9Z1Q5	Thyme/10
Heterogeneous nuclear ribonucleoprotein U	HNRNPU	0.008	0.51	NEIL1-mediated oxidative repair	Q8VEK3	Thyme/10
Isoform 2 of Aspartyl/asparaginyl beta-hydroxylase	ASPH	< 0.0001	-0.99	Cardiac contraction	Q8BSY0	Thyme/10
Tropomyosin beta chain	TPM2	0.01	-0.79	Cardiac contraction	P58774	Thyme/10
Tropomyosin alpha-1 chain	TPM1	0.006	-0.52	Cardiac contraction	P58771	Thyme/10
Transforming protein RhoA	RHOA	0.04	0.69	Cardiac contraction	Q9QUI0	Thyme/10
Nuclease-sensitive element-binding protein 1	YBX1	0.03	-1.51	NEIL2-mediated repair under oxidative stress	P62960	Thyme/20
Acidic leucine-rich nuclear phosphoprotein 32 family member A	ANP32A	< 0.0001	-1.13	Oxidative stress regulation via ATM	O35381	Thyme/20

Protein MEMO1	MEMO1	0.02	-1.07	NADH oxidase-induced redox signaling	Q91VH6	Thyme/20
Hydroxyacylglutathione hydrolase, mitochondrial	HAGH	0.02	-0.96	Detoxification of methylglyoxal	Q99KB8	Thyme/20
Fructose-2,6-bisphosphatase TIGAR	TIGAR	0.03	-0.83	ROS levels regulation	Q8BZA9	Thyme/20
28S ribosomal protein S36, mitochondrial	MRPS36	< 0.0001	-0.81	Tricarboxylic acid cycle	Q9CQX8	Thyme/20
Isoform 2 of Mitochondrial import inner membrane translocase subunit Tim21	TIMM21	0.02	-0.81	Mitochondrial respiratory chain assembly	Q8CCM6	Thyme/20
Ribosome-recycling factor, mitochondrial	MRRF	0.04	-0.63	Mitochondrial translational machinery	Q9D6S7	Thyme/20
Heterogeneous nuclear ribonucleoprotein D0	HNRNPD	0.005	-0.54	Elimination of oxidized RNA	Q60668	Thyme/20
Cytochrome c1, heme protein, mitochondrial	CYC1	0.02	-0.54	Mitochondrial respiratory chain	Q9D0M3	Thyme/20
NADH dehydrogenase [ubiquinone] 1 beta subcomplex subunit 6	NDUFB6	0.02	-0.54	Mitochondrial respiratory chain	Q3UIU2	Thyme/20
Mitochondrial-processing peptidase subunit beta	PMPCB	< 0.0001	-0,52	Peroxisomal lipid metabolism	Q9CXT8	Thyme/20
Cytochrome c oxidase assembly factor 7	COA7	0.04	1.02	Mitochondrial respiratory chain assembly	Q921H9	Thyme/20
ADP/ATP translocase 4	SLC25A31	0.02	0.97	Mitochondrial ADP:ATP antiporter and proton transporter	Q3V132	Thyme/20
Heat shock protein 75 kDa, mitochondrial	TRAP1	0.03	0.77	Maintaining mitochondrial function and polarization	Q9CQN1	Thyme/20
Chloride intracellular channel protein 1	CLIC1	0.007	0.77	ROS generation by the NAPDH oxidase	Q9Z1Q5	Thyme/20
Clusterin	CLU	0.02	0.66	Oxidative stress-induced cell death regulation	Q06890	Thyme/20

Ras-related protein Rab-5C	RAB5C	0.02	0.55	Oxidative stress response	P35278	Thyme/20
Isoform 2 of Aspartyl/asparaginyl beta-hydroxylase	ASPH	< 0.0001	-1.11	Cardiac contraction	Q8BSY0	Thyme/20
Tropomyosin beta chain	TPM2	0.006	-0.87	Cardiac contraction	P58774	Thyme/20
Ras GTPase-activating-like protein IQGAP1	IQGAP1	0.01	-1.56	ROS regulation through Nrf2	Q9JKF1	Oregano/10
Small ubiquitin-related modifier 3	SUMO3	0.003	-1.49	SUMOylation, process regulated by cellular oxidative stress	Q9Z172	Oregano/10
Acyl carrier protein, mitochondrial	NDUFAB1	0.001	-1.23	Mitochondrial respiratory chain	Q9CR21	Oregano/10
NEDD8-activating enzyme E1 regulatory subunit	NAE1	0.02	-1.11	Promotes oxidative metabolism in heart	Q8VBW6	Oregano/10
Ribosome-recycling factor, mitochondrial	MRRF	0.02	-0.97	Mitochondrial translational machinery	Q9D6S7	Oregano/10
Delta-1-pyrroline-5-carboxylate dehydrogenase, mitochondrial	ALDH4A1	0.03	-0.89	Carbonyl metabolism	Q8CHT0	Oregano/10
Mitogen-activated protein kinase 3	MAPK3	0.002	-0.82	ROS regulation through Nrf2	Q63844	Oregano/10
MICOS complex subunit MIC19	CHCHD3	0.02	-0.72	Oxidative stress response	Q9CRB9	Oregano/10
Isoform 2 of Mitochondrial import inner membrane translocase subunit Tim21	TIMM21	0.02	-0.69	Mitochondrial respiratory chain assembly	Q8CCM6	Oregano/10
Glutathione S-transferase Mu 5	GSTM5	0.01	-0.67	Glutathione conjugation	P48774	Oregano/10
CDGSH iron-sulfur domain-containing protein 2	CISD2	0.01	-0.65	Iron metabolism	Q9CQB5	Oregano/10
Alcohol dehydrogenase [NADP(+)]	AKR1A1	0.03	-0.61	Carbonyl metabolism	Q9JII6	Oregano/10
Cytochrome b5 type B	CYB5B	0.01	-0.53	Nitric oxide biosynthesis and electron carrier for oxygenases	Q9CQX2	Oregano/10

Elongation factor Tu, mitochondrial	TUFM	0.03	-0.53	Protein translation in mitochondria	Q8BFR5	Oregano/10
Heterogeneous nuclear ribonucleoprotein D0	HNRNPD	< 0.0001	-0.53	Elimination of oxidized RNA	Q60668	Oregano/10
Mitochondrial import receptor subunit TOM22 homolog	TOMM22	0.006	-0.52	Electron carrier for oxygenases	Q9CPQ3	Oregano/10
Perilipin-4	PLIN4	0.01	1.3	Lipid metabolism	O88492	Oregano/10
Mitochondrial coenzyme A transporter SLC25A42	SLC25A42	0.01	0.91	Mitochondrial carrier mediating the transport of coenzyme A	Q8R0Y8	Oregano/10
Myoglobin	MB	0.01	0.68	Oxygen reserve and nitric oxide regulation	P04247	Oregano/10
Acyl-CoA synthetase family member 2, mitochondrial	ACSF2	0.02	0.64	Lipid metabolism and fatty acid oxidation	Q8VCW8	Oregano/10
Clusterin	CLU	0.03	0.64	Oxidative stress-induced cell death regulation	Q06890	Oregano/10
PGC-1 and ERR-induced regulator in muscle protein 1	PERM1	0.04	0.62	Mitochondrial biogenesis and oxidative capacity	Q149B8	Oregano/10
NADH dehydrogenase [ubiquinone] 1 alpha subcomplex subunit 5	NDUFA5	0.03	0.6	Mitochondrial respiratory chain	Q9CPP6	Oregano/10
Trans-1,2-dihydrobenzene-1,2-diol dehydrogenase	DHDH	0.002	0.53	NADP1-linked oxidation of transdihydrodiols of aromatic hydrocarbons	Q9DBB8	Oregano/10
Peroxiredoxin-6	PRDX6	0.04	0.5	Redox regulation	O08709	Oregano/10
Isoform 5 of LIM domain-binding protein 3	LDB3	< 0.0001	-1.81	Cardiac contraction	Q9JKS4	Oregano/10
Ras GTPase-activating-like protein IQGAP1	IQGAP1	0.01	-1.56	Cardiac contraction	Q9JKF1	Oregano/10
Homeodomain-only protein	HOPX	< 0.0001	-1.3	Cardiac contraction	Q8R1H0	Oregano/10
Isoform 2 of Aspartyl/asparaginyl beta-hydroxylase	ASPH	< 0.0001	-1.04	Cardiac contraction	Q8BSY0	Oregano/10

Isoform 2 of Tropomyosin alpha-3 chain	TPM3	0.03	-0.85	Cardiac contraction	P21107	Oregano/10
Actin-related protein 2	ACTR2	0.03	-0.78	Cardiac contraction	P61161	Oregano/10
Myosin-1	MYH1	0.01	1.44	Cardiac contraction	Q5SX40	Oregano/10
Dystrobrevin alpha	DTNA	0.04	1.33	Cardiac contraction	Q9D2N4	Oregano/10
Coiled-coil-helix-coiled-coil-helix domain-containing protein 2	CHCHD2	0.01	-1.87	Oxidative stress response	Q9D1L0	Oregano/20
Small ubiquitin-related modifier 3	SUMO3	0.002	-1.64	SUMOylation, process regulated by cellular oxidative stress	Q9Z172	Oregano/20
NADH-ubiquinone oxidoreductase chain 4	MTND4	0.04	-1.34	Mitochondrial respiratory chain	P03911	Oregano/20
Protein-glutamine gamma-glutamyltransferase 2	TGM2	0.04	-0.74	Promote apoptosis under oxidative stress	P21981	Oregano/20
NADH dehydrogenase [ubiquinone] 1 beta subcomplex subunit 5, mitochondrial	NDUFB5	0.02	-0.67	Mitochondrial respiratory chain	Q9CQH3	Oregano/20
Mitochondrial import inner membrane translocase subunit TIM16	PAM16	0.04	-0.6	Reactive oxygen species (ROS) homeostasis	Q9CQV1	Oregano/20
Lipoamide acyltransferase component of branched-chain alpha-keto acid dehydrogenase complex, mitochondrial	DBT	0.04	-0.51	Inner-mitochondrial enzyme complex	P53395	Oregano/20
Fumarylacetoacetate hydrolase domain-containing protein 2A	FAHD2A	0.03	-0.51	Hydrolase activity mitochondrial enzyme	Q3TC72	Oregano/20
Cytochrome b5 type B	CYB5B	0.01	-0.51	Nitric oxide biosynthesis and electron carrier for oxygenases	Q9CQX2	Oregano/20
Carbonic anhydrase 1	CA1	0.01	2.19	Hydration of carbon dioxide	P13634	Oregano/20
Hemoglobin subunit beta-2	HBB-B2	0.02	1.85	Oxygen transport	P02089	Oregano/20
Bisphosphoglycerate mutase	BPGM	0.01	1.78	Hemoglobin oxygen affinity regulation	P15327	Oregano/20

Thiosulfate sulfurtransferase	TST	0.004	1.66	Sulfur metabolism and detoxification and anti-oxidative stress functions	P52196	Oregano/20
Hemoglobin subunit beta-1	HBB-B1	0.006	1.6	Oxygen transport	P02088	Oregano/20
Hemoglobin subunit alpha	HBA	0.04	1.34	Oxygen transport	P01942	Oregano/20
Carbonic anhydrase 3	CA3	0.009	1.31	Hydration of carbon dioxide	P16015	Oregano/20
Hemopexin	HPX	0.01	1.09	Heme transport and oxidative stress protection	Q91X72	Oregano/20
Clusterin	CLU	0.007	0.82	Oxidative stress-induced cell death regulation	Q06890	Oregano/20
Dehydrogenase/reductase SDR family member 11	DHRS11	0.05	0.79	Reduce alpha-dicarbonyl compounds	Q3U0B3	Oregano/20
obg-like ATPase 1	OLA1	0.01	0.76	Oxidative stress response	Q9CZ30	Oregano/20
Glutathione peroxidase 1	GPX1	0.01	0.68	Hydrogen peroxide degradation	P11352	Oregano/20
Serotransferrin	TF	0.003	0.63	Iron metabolism	Q921I1	Oregano/20
Catalase	CAT	0.03	0.56	Hydrogen peroxide degradation	P24270	Oregano/20
Ras-related protein Rab-5C	RAB5C	0.01	0.54	Oxidative stress response	P35278	Oregano/20
Trans-1,2-dihydrobenzene-1,2-diol dehydrogenase	DHDH	0.003	0.5	NADP1-linked oxidation of transdihydrodiols of aromatic hydrocarbons	Q9DBB8	Oregano/20
Isoform 5 of LIM domain-binding protein 3	LDB3	0.009	-0.95	Cardiac contraction	Q9JKS4	Oregano/20
Tropomyosin alpha-4 chain	TPM4	0.005	1.3	Cardiac contraction	Q6IRU2	Oregano/20
Calsequestrin-2	CASQ2	0.04	0.99	Cardiac contraction	O09161	Oregano/20

GeneCards database (http://www.genecards.org/) was used to identify the biological function of the proteins.
